# Effect of an NGR Peptide on the Efficacy of the Doxorubicin Phospholipid Delivery System

**DOI:** 10.3390/nano13152229

**Published:** 2023-08-01

**Authors:** Lyubov V. Kostryukova, Yulia A. Tereshkina, Elena G. Tikhonova, Yulia Yu. Khudoklinova, Daria V. Bobrova, Alisa M. Gisina, Galina E. Morozevich, Veronica V. Pronina, Tatiana V. Bulko, Victoria V. Shumyantseva

**Affiliations:** Institute of Biomedical Chemistry, 10 Pogodinskaya St., 119121 Moscow, Russia; kostryukova87@gmail.com (L.V.K.); burova13@gmail.com (Y.A.T.); elena.tikhonova_@mail.ru (E.G.T.); dariansay@mail.ru (D.V.B.); alisa.gisina@gmail.com (A.M.G.); galina.morozevich@ibmc.msk.ru (G.E.M.); veronicapunch@mail.ru (V.V.P.); tanyabulko@mail.ru (T.V.B.); viktoria.shumyantseva@ibmc.msk.ru (V.V.S.)

**Keywords:** phospholipid nanoparticles, targeted drug delivery, tumor, doxorubicin, NGR, aminopeptidase N/CD13, cytotoxicity assay, apoptosis, electrostatic interactions

## Abstract

This study is a continuation of an investigation into the effect of a targeted component, a peptide with an NGR, on the properties of the previously developed doxorubicin phospholipid delivery system. The NGR peptide has an affinity for aminopeptidase N (known as the CD13 marker on the membrane surface of tumor cells) and has been extensively used to target drug delivery systems. This article presents the results of a study investigating the physical properties of the phospholipid composition with and without the peptide chain: particle size, zeta potential, stability in fluids, and dependence of doxorubicin release from nanoparticles at different pH levels (5.0, 6.5, 7.4). The cytotoxic effect of the compositions has also been shown to depend on the dose of the drug used for incubation, the presence of the targeted component in the composition, and the time of incubation time of the substances. There was a significant difference in the cytotoxic effect on HT-1080 (CD13-positive) and MCF-7 (CD13-negative) cells. Cell death pathway analysis has shown that death occurred mainly by apoptosis. We also present data on the effect of doxorubicin embedded in phospholipid nanoparticles with the targeted peptide on DNA assessed by differential pulse voltammetry, the mechanism of action being electrostatic interactions. The interactions of native dsDNA with doxorubicin encapsulated in phospholipid nanoparticles with the targeted peptide were studied electrochemically by differential pulse voltammetry. Here, we have highlighted that the targeted peptide in the doxorubicin composition moved specific interaction of the drug with dsDNA from intercalative mode to electrostatic interactions.

## 1. Introduction

**Scheme 1:**
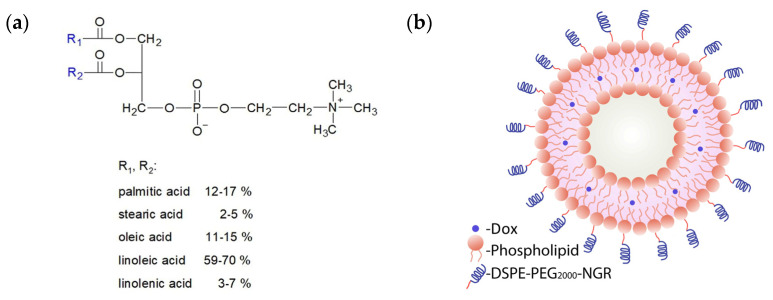
Structure of phosphatidylcholine (**a**) and model of Dox incorporated to a phospholipid system with peptides (**b**).

Cancer is the second leading cause of death (after cardiovascular disease) worldwide, having accounted for nearly 10 million deaths in 2020 [1,2]. Doxorubicin (Dox) is an effective anticancer drug used to treat solid tumors, leukemia, and lymphoma [3]. One of the mechanisms of action of Dox is the intercalation between DNA nucleobases leading to a strong interaction between DNA and the anthracycline ring and disrupting DNA synthesis and transcription [4,5]. Dox is known to have a high binding constant to double-stranded DNA (~10^6^ M^−1^), confirming intercalation mode [6]. However, the therapeutic concentrations of Dox are limited by its adverse dose-dependent cardiotoxicity [7]. Nanotechnology is a promising alternative to overcome this limitation of Dox in cancer therapy. Drug content may be increased in local area using nanoparticles (NPs; diameter 1–100 nm) [8]. Liposomes formed by one or more lipid bilayers are highly effective as drug delivery vehicles, including intra-articular injection [9]. Liposomal encapsulation makes it possible to change the tissue distribution and pharmacokinetics, and increase the therapeutic index of conventional Dox. The size of drug-encapsulated liposomes ensures extravasation through tumor tissue vessels, while penetration into dense connective tissue, such as heart cells, is hindered [10]. Moreover, drug encapsulation in NPs prevents or reduces their enzymatic degradation [11]. Of particular interest are nanoparticles, including liposomes, based on plant phospholipids, a natural “building material” and a component of all biological membranes. Phospholipids, compared to various synthetic materials, are biodegradable, biologically inert, and do not cause allergic, antigenic, or pyrogenic reactions; i.e., they are safe for pharmaceutical use [12]. We previously published material on the development of a phospholipid transport system [13]. Phosphatidylcholine structures and a model of Dox incorporated to a phospholipid system with peptides are presented below in Scheme 1.

To increase bioavailability and reduce side effects, NPs are modified with various targeted molecules: peptides [14,15,16], aptamers [17,18,19], small molecules [20,21,22], and antibodies [23,24]. The concept of selective drug targeting is based on the high expression of certain cell surface components in tumors or newly formed tumor vessels [25]. Solid tumors are known to recruit new blood vessels to support tumor growth, so angiogenic tumor vessels can also be used in clinical practice to develop better targeted therapies [26]. One cancer-specific receptor is aminopeptidase N (APN/CD13), which is a multifunctional glycoprotein that acts as a peptidase, receptor, and signaling molecule in some tissues. APN activity has been associated with progression of many types of cancer, indicating significant therapeutic potential for the treatment of cancer [27]. The NGR peptide (with the asparagine-glycine-arginine amino acid sequence) targets the APN isoform expressed on endothelial cells in angiogenic vessels, such as new vessels in tumors. APN/CD13 is widely accepted as a rational target for therapeutic development due to its accessible location (i.e., tumor vasculature) [28]. The peptide with the NGR motif (Asn-Gly-Arg) with a disulfide bridge between cys1 to cys5 is known to exhibit strong cytotoxicity and activity against tumor cells due to the fact it binds with CD13 on tumor cells [29]. This peptide shows dose-dependent antiproliferation against tumor cells and induces cell cycle arrest at G2/M phases and apoptosis of the tumor cells. NGR motif-containing peptides are useful in delivering cytotoxic drugs, and are actively used as drug delivery systems to the tumor vasculature.

The high ligand specificity makes NGR a suitable candidate for the active targeting of macromolecular or nanoparticle systems in vivo.

We have previously developed a Dox phospholipid composition (NPh-Dox) and studied its efficacy in vivo [30,31]. The inclusion of Dox in phospholipid nanoparticles contributed to its accumulation in the tumor and led to an increase in its specific activity. In subsequent experiments, the resulting Dox phospholipid composition was constructed using a targeted NGR peptide (NPh-Dox-NGR) [32]. This work is a comprehensive study of the physical properties of the Dox phospholipid composition with and without the targeted ligand in comparison with free Dox (such as particle size, degree of release from nanoparticles depending on the pH of the medium, and changes in the size and distribution of particles during incubation in isotonic solutions), in vitro cytotoxicity of these forms of Dox, cell death pathways after incubation with Dox products, and their interaction with double-stranded DNA (dsDNA). We herein present the first study on the comparative effects of Dox, NPh-Dox, and Dox with NGR peptide (NPh-Dox-NGR) on subsequent DNA–drug interaction.

## 2. Materials and Methods

### 2.1. Materials

The drug substance of doxorubicin with a 99% g/c purity was obtained at the Omutninskaya Scientific Pilot-Industrial Base (JSC Omutninsk Scientific Experimental-Industrial Base, Vostochnyi, Russia). Soybean phosphatidylcholine Lipoid S100 (Lipoid, Ludwigshafen, Germany) was used as the raw material for the nanoparticles. DSPE-PEG(2000)–Maleimide for attaching the targeted peptide was obtained by Avanti Lipids (Avanti, Alabaster, AL, USA). The targeted peptide NGR ((NH_2_-)Gly-Asn-Gly-Arg-Gly-Cys(-COOH)) with a purity of at least 95%, as confirmed by HPLC with MS detection and NMR spectroscopy, was obtained at Sinton-Lab LLC (Sinton-Lab LLC, St. Petersburg, Russia). NaCl, D(+)-Glucose produced by Merck (Merck, Darmstadt, Germany), phosphate-buffered saline (PBS) (PanEco LLC, Moscow, Russia), and ethyl alcohol (96% purity) from Medkhimprom (Medkhimprom LLC, Balashikha, Russia) were also used in the study. Distilled water was prepared using a GFL-2004 water distiller (GFL, Burgwedel, Germany).

Cell cultures were incubated using DMEM culture media, Versene solution (PanEco LLC., Moscow, Russia), and fetal bovine serum (FBS) (Gibco, Gaithersburg, MD, USA).

The survival of tumor cells was evaluated using the MTT test (Sigma, Darmstadt, Germany). Cell death was assessed using a special FITC Annexin V Apoptosis Detection Kit I (BD Biosciences, Franklin Lakes, NJ, USA) consisting of FITC-conjugated Annexin V, propidium iodide (PI), and annexin-binding buffer.

### 2.2. Methods

#### 2.2.1. Cell Line

We used cell lines of human fibrosarcoma (cell culture HT-1080) and breast cancer (MCF-7) obtained at the Research Institute of Virology (D. I. Ivanovsky Institute of Virology, Moscow, Russia). Tumor cells were cultured according to the recommendations specified in the cell culture certificates. Cells were incubated in DMEM culture media supplemented with 2 mM L-glutamine and 10% FBS in 25 cm^2^ and 75 cm^2^ culture flasks (Corning, Glendale, AZ, USA) at 37 °C in a humid atmosphere with 5% CO_2_ in a Sanyo CO_2_ incubator (Sanyo, Moriguchi, Japan). Cell lines were used in the study after 3 to 10 passages.

#### 2.2.2. Preparation of Doxorubicin Compositions

The DSPE-PEG(2000)–NGR conjugate was obtained according to the method described earlier [33]. To obtain a composition with a targeted fragment (NPh-Dox-NGR), the Lipoid S100 phospholipid was dissolved in 96% ethanol at a ratio of 125:1 (*w*/*w*); the DSPE-PEG(2000)–NGR conjugate was separately dissolved in 96% ethanol at a ratio of 25:1 (*w*/*w*). The obtained alcohol solutions were mixed in the Lipoid:conjugate at a 10:1 (*v*/*v*) ratio. The alcohol was removed with a Heidolph Laborota 4003 rotary evaporator (Heidolph, Schwabach, Germany) until a dry film was obtained under the following conditions: 60 mbar, water temperature 30 °C, 1190 rpm, for 8–10 min. The resulting film was rehydrated with an aqueous solution of Dox (Dox:Lipoid 1:20 ratio (*w*/*w*)). The coarse emulsion was processed on a Bandelin Sonopuls ultrasonic disintegrator (Bandelin, Düsseldorf, Germany) using a KE72 titanium rod for 6 min at a power of 50%. The comparator (NPh-Dox nanoparticles) was prepared in the same way using only soy phosphatidylcholine (Lipoid S100) and Dox (film method) as described above. The Dox:Lipoid ratio was 1:20 (*w*/*w*).

The resulting doxorubicin compositions were analyzed by particle size, zeta potential using Zetasizer Malvern ZS (Malvern Instruments Ltd., Malvern, UK) substance concentration, and percentage of incorporation into nanoparticles (Agilent 1100 Series Liquid Chromatograph (Agilent Technology, Santa Clara, CA, USA) with a diode-matrix detector and ChemStation Rev. A.09.03 software).

#### 2.2.3. Stability in NaCl and Glucose Solutions

A volume of 0.5 mL of each sample of the Dox phospholipid composition (with and without the target peptide) was incubated in 0.5 mL of 1.8% NaCl solution or 10% glucose at 37 °C in a water bath. In each sample, at the starting point and after 0.25, 0.5, 1, 2, 5, 24, 48, and 72 h, we controlled the particle size and their distribution using the Zetasizer Malvern ZS.

#### 2.2.4. Release of Doxorubicin from Phospholipid Nanoparticles

The release of Dox from NPs (with and without the target peptide) was evaluated using dialysis bags (3.5 kDa cut-off threshold). Briefly, 1 mL of test samples (Dox concentration was 1 mg/mL) in the dialysis bags were placed in 25 mL of PBS (pH 7.4, 6.5 and 5.0), then incubated and mixed at 37 °C and 100 rpm in a Grant OLS 200 shaker water bath (Grant Instruments (Cambridge) Ltd., Shepreth, UK). The aliquots of the supernatant (1 mL) were sampled at certain intervals for each variant (0.25, 0.5, 1, 3, 24, 48, and 72 h). After each sampling, an equal amount of PBS was added. The concentration was determined using a spectrophotometer Agilent 8453 spectrophotometer (Agilent Technologies, Waldbronn, Germany) at a wavelength of 254 nm. The rate of drug release was calculated by dividing the concentration of each drug (released from drug in phospholipid nanoparticle) at a given time by the initial concentration of the corresponding drug in the phospholipid nanoparticle [34].

#### 2.2.5. Stability at Different Values of pH

Samples of compositions NPh-Dox and NPh-Dox-NGR were diluted 10-fold with PBS with the appropriate pH environment (7.4, 6.5 and 5.0). Solutions were incubated at 37 °C. The particle size was measured on the Zetasizer Malvern ZS device at certain time intervals (0, 0.25, 0.5, 1, 2, 5, 24, and 48 h).

#### 2.2.6. In Vitro Cytotoxicity Assay

The cytotoxicity of the developed Dox compositions was evaluated in vitro using the MTT test. HT-1080 cells (10^5^ cells per well) were seeded into sterile 96-well culture plates and incubated at 37 °C in 5% CO_2_ using a Binder CO_2_ incubator (Binder, Tuttlingen, Germany) for 24–26 h. Then, the test substances were added at Dox concentrations of 25, 10, 5, 1, 0.1, and 0 µM and the cells were incubated for 24 h and 48 h.

After that, 20 µL of MTT (5 mg/mL) was carefully added to each well and incubated at 37 °C for 4 h. The medium was then carefully removed and 150 µL of MTT solvent was added. The cells were covered with foil and shaken on an orbital shaker for 15 min. The absorbance was recorded at 550 nm using Multiscan FC (ThermoSpectronic, Waltham, ML, USA) and normalized to the untreated control (without Dox).

#### 2.2.7. Cell Death Assay

The cells were cultured in 6-well plates until the monolayer reached 80–90% confluence. Then the studied samples of doxorubicin (Dox, NPh-Dox, and NPh-Dox-NGR) were added to the medium with Dox concentrations of 0.2 µM, 1 µM, and 2.0 µM and incubated for 24 h (37 °C, 5% CO_2_). Next, the cells were washed from the medium twice with Hanks’ solution, trypsin and Versene solutions (1:1) were added, and the resulting solution was suspended by pipetting. Then the cells were washed once in Hanks’ solution by centrifugation at 1200 rpm using Centrifuge 5810R (Eppendorf, Hamburg, Germany) for 5 min. The resulting sediment was resuspended in 100 µL of binding buffer. The cells were incubated with 5 µL of Annexin V-FITC and 5 µL of PI in a dark place for 15 min at room temperature. Cell staining was analyzed using a FACSAria III flow cytometer sorter (BD Biosciences, Franklin Lakes, New Jersey, USA) equipped with blue (488 nm) and yellow-green (561 nm) lasers. The results were analyzed using BD FACSDiva Software version 7. The graphical representation of the data was produced using FlowJ version 10.

#### 2.2.8. Electrochemical Study of Dox Interaction with DNA

Three-pronged screen-printed electrodes (SPEs) purchased from Color Electronics (Moscow, Russia, http://www.colorel.ru, assessed on 26 July 2023) were used for the electrode preparation. They consist of a round graphite working electrode (2 mm in diameter) surrounded by a graphite ringed auxiliary counter-electrode and an Ag/AgCl reference electrode. Electrochemical measurements were performed on an Autolab 302N potentiostat/galvanostat (Metrohm Autolab, Utrecht, The Netherlands) with Nova software (version 2.0). The electrodes were modified with 2 μL of a freshly prepared dispersion of 1 mg/mL single-walled carbon nanotubes stabilized with carboxymethyl cellulose (SPE/CNT) and dried at room temperature for 25 min [35].

Fish-sperm double-stranded DNA (dsDNA, as lyophilized powder) was obtained from Sigma-Aldrich (Sigma-Aldrich, St. Louis, MO, USA). The purity of the DNA stock solution was confirmed by taking the absorbance ratio of A_260_/A_280_, which was found to be in the range of 1.8–1.9, indicating the lack of contamination of protein in the dsDNA. The stock solution of dsDNA (1.5 mg/mL) was prepared in a 100 mM potassium phosphate buffer with 50 mM NaCl (pH 7.4). The Dox concentrations were 5 µM. The measurements were carried out horizontally with a 60 μL drop at room temperature 23 °C in 100 mM potassium phosphate buffer (pH 7.4) with 50 mM NaCl as a supporting electrolyte by differential pulse voltammetry (DPV). The DPV parameters were: potential range: 0.2–1.2 V, pulse amplitude: 0.025 V, potential step: 0.005 V, pulse duration: 50 ms, modulation amplitude: 0.05 V. The dsDNA–drug complexes were incubated for 10 min. All potentials were referred to the Ag/AgCl reference electrode.

#### 2.2.9. Statistical Analysis

All experiments were conducted three times. The article presents the data as an average value ± the standard error of the mean. The differences were considered significant at *p* < 0.05.

## 3. Results

### 3.1. Physical Properties of Phospholipid Compositions NPh-Dox and NPh-Dox-NGR

**Figure 1:**
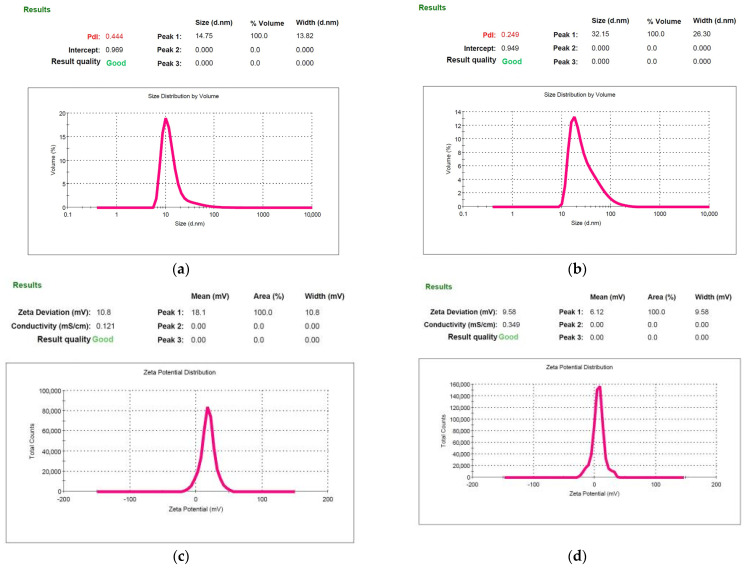
Particle size of aqueous solutions of compositions (**a**) NPh-Dox; (**b**) NPh-Dox-NGR. ζ-potential: (**c**) NPh-Dox; (**d**) NPh-Dox-NGR.

The results of the influence of the targeted peptide with the NGR incorporated into the Dox phospholipid composition on the particle size and electrokinetic (ζ-) potential are shown in Figure 1.

In the phospholipid composition of Dox without the target fragment, the particle size was 14.75 ± 0.37 nm (97%). The presence of the targeted fragment in the form of DSPE-PEG(2000)–NGR led to an almost twofold enlargement of particles; their size was 32.15 ± 2.46 nm (92% of the particles).

The values of the ζ-potential were positive for all variants of Dox compositions. In particular, the ζ-potential was 16.1 ± 1.2 mV for the phospholipid composition (NPh-Dox) and 6.12 ± 1.2 mV for the sample with the targeted peptide (Figure 1). The percentage of Dox incorporation into nanoparticles in both cases was 98 ± 1.1%.

### 3.2. Stability of Dox Compositions in NaCl and Glucose Solutions

The stability of the Dox compositions was evaluated in isotonic solutions (0.9% NaCl and 5% glucose). The study showed that the particle size at the starting point in the NPh-Dox sample increased almost 2.3-fold from the initial size after incubation in 0.9% NaCl (Table 1). Further incubation had practically no effect on the particle size (about 29.94 ± 1.69 nm), and the distribution varied from 99.9 to 96.57% in terms of volume. Incubation in 5% glucose led to a slight increase in particle size by 2–4 nm; after 3 days of incubation, the size did not change.

In the sample with the targeted peptide (NPh-Dox-NGR), the particle size differed slightly from the initial one (by 4–5 nm) after incubation in 0.9% NaCl. After 3 days, no significant changes in particle size were observed; the distribution had decreased by 5%. During incubation of NPh-Dox-NGR in 5% glucose, no changes in particle size were observed compared to the initial solution.

Therefore, these experiments confirmed the stability of drug formulations of Dox in physiologically relevant solutions, such as 0.9% NaCl or 5% glucose.

### 3.3. Release of Doxorubicin from Nanoparticles Depending on pH

**Figure 2:**
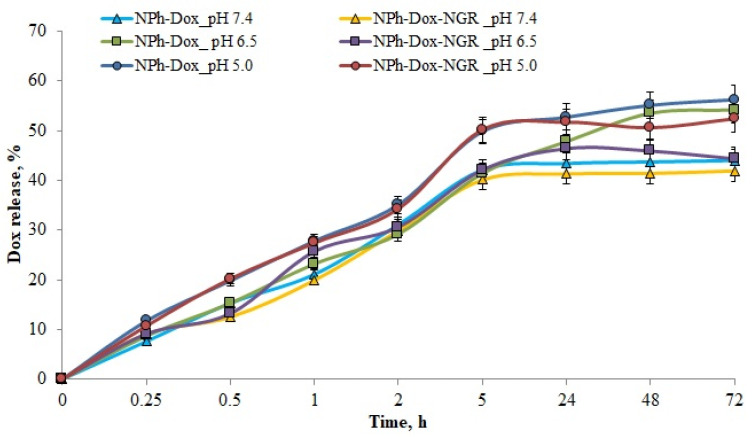
Release of Dox from NPs of NPh-Dox and NPh-Dox-NGR compositions during incubation in solutions with different pH values (5.0, 6.5 and 7.4).

The natural pH gradient is 5.0–6.5 in endosomes or lysosomes of tumor cells, and 6.5–7.2 in the tumor microenvironment [36]. Therefore, the conditions of drug release into the bloodstream and intracellular compartments of tumor cells in this experiment were simulated using pH 7.4, 6.5, and pH 5.0 buffer solutions. The results of Dox release from NPh-Dox-NGR and NPh-Dox NP compositions are shown in Figure 2.

It was shown that an increase in the acidity of the medium (pH 6.5 and 5.0) led to an increase in the release of Dox (from 40 to 55%) during incubation for 5 h or more. The percentage of Dox release for NPh-Dox after 72 h was 44% at pH 7.4 and 56% at pH 5.0. It should be noted that there were no differences in the degree of drug release at pH 7.4 depending on the presence of the targeted peptide in the composition. However, at pH 6.5 the release of the drug in the variant without peptides was greater than 10% more compared to the formulation containing NGR. After 5 h of incubation at pH 7.4, for both variants of the phospholipid compositions, regardless of the presence of the peptide, doxorubicin was released almost equally and remained at this level up to 72 h. It should be noted that at pH 5.0, the drug release values were almost 10% higher than at pH 7.4 for both variants (with and without peptide).

### 3.4. Stability of Dox Compositions at Different Values of pH

**Figure 3:**
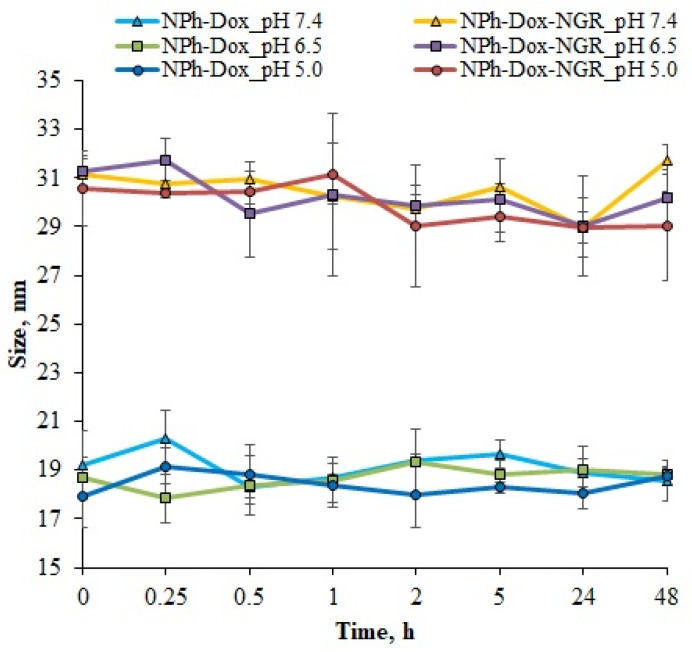
Stability of Dox compositions at different values of pH.

Figure 3 shows the stability results of NPh-Dox and NPh-Dox-NGR compositions during incubation in different values pH (7.4, 6.5 and 5.0).

It has been shown that different pH values do not affect the particle size and agglomeration.

### 3.5. In Vitro Cytotoxicity

**Figure 4:**
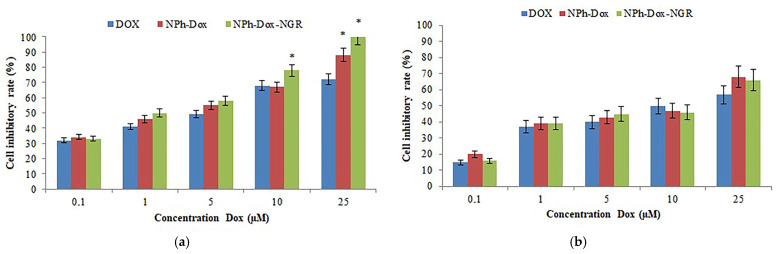
Growth inhibition of HT-1080 (**a**) and MCF-7 (**b**) cells after incubation for 24 h. * *p* < 0.05 (*n* = 3).

Previously [37], we have shown that the NGR marker, aminopeptidase N/CD13, is expressed on the surface of HT-1080 (CD13-positive) fibrosarcoma cells, while MCF-7 cells are not positive in terms of CD13 expression and were chosen as a negative control (CD13-negative). Therefore, these cell lines were also selected in this experiment. They were treated with increasing doses of Dox for 24 and 48 h and the metabolic activity was quantified by MTT assay (Figure 4).

When incubated for 24 h, the cytotoxic effect of Dox was dose-dependent. The Dox concentration of 25 µM resulted in 100% death of HT-1080 cells when incubated with NPh-Dox-NGR; for free Dox, cell growth inhibition was about 70%. For the Dox concentration of 10 µM, the degree of dead cells during incubation with the composition containing the targeted peptide was 10% higher than the degree for the free substance and NPh-Dox. At the minimum concentration of 0.1 µM, the effect of the test samples was practically the same, within the error range. At other concentrations, the three variants showed close results; however, the cytostatic effect of the NPh-Dox-NGR composition was significantly higher (by 9%) in comparison with the free drug. When the Dox compositions were incubated with HT-1080 cells for 48 h, 100% cell death was observed in all samples.

In the MCF-7 cell line not expressing CD13, cell growth inhibition was lower compared to HT-1080 for all samples. After 24 h of incubation with the substances, there was also a direct dependence of the manifestation of the cytotoxic effect on the studied doses. At the same time, there was no significant difference between the samples with free Dox embedded in NPs and those in the composition with the targeted peptide. With an increase in the time of incubation of cells with the Dox compositions and free substance up to 48 h, there was an increase in tumor cell growth inhibition up to 75–78% regardless of the drug dose used, except for the maximum one. At the maximum concentration (25 µM), Dox embedded in NPs “killed” 100% of the cells, while the free one “killed” 68%.

It is also worth noting that in vitro experiments that we have previously carried out have demonstrated decreased drug association with blood cells for this nanophospholipid form as compared with free doxorubicin. This was accompanied by a corresponding increase in its plasma level and by drug redistribution from plasma protein fraction to high density lipoproteins [38].

### 3.6. Apoptosis Induction in the HT-1080 Cell Line

**Figure 5:**
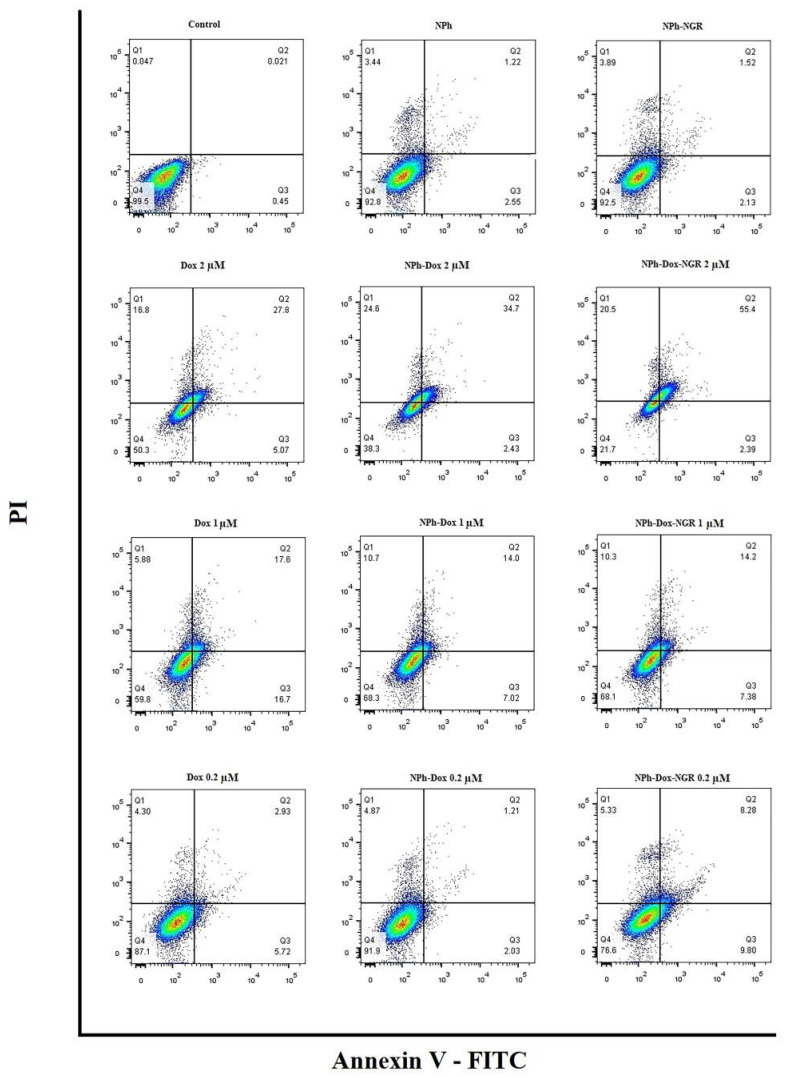
Analysis of apoptosis in HT-1080 cell culture incubated with phospholipid NPs (NPh), phospholipid NPs with the targeted peptide (NPh-NGR); free doxorubicin (Dox); phospholipid composition (NPh-Dox); and phospholipid composition with the peptide (NPh-Dox-NGR). Dox concentrations 2 µM, 1 µM, 0.2 µM. Quadrant design: upper left (Q1): necrosis, cells stained with PI; top right (Q2): late apoptosis; bottom right (Q3): early apoptosis, cells stained with annexin V; bottom left (Q4): fluorescent signal at the autofluorescence level of unstained cells.

The assessment of cell death after 24 h of incubation with Dox-containing compositions was analyzed using flow cytometry. The results obtained are shown in Figure 4. To understand the effect of all components of the system on cell death, we studied the effect of free Dox, the phospholipid system without an embedded drug and without the peptide (NPh). The concentration of phospholipids and NGR (for NPh-NGR or NPh) was taken to be the same as for the composition NPh-Dox-NGR with a Dox concentration of 2 µM. As a result, it was shown that less than 8% of cells died (less than 3.9% of them were cells exhibiting necrosis) with the NPh and NPh-NGR systems (Figure 5).

When the cells were incubated in variants with a Dox concentration of 2 µM or more for 24 h, death was predominantly through apoptosis (however, necrosis was observed in less than 25% of cells). An increase in Dox concentration was shown to shift the population towards late apoptosis. The percentage of cells undergoing apoptosis varied from 8.3% (0.2µM NPh-Dox-NGR) to 55.4% (2 µM NPh-Dox-NGR). Late apoptosis was also shown to be more pronounced after incubation with Dox compositions containing the targeted peptide. At the minimum concentration studied for Dox (0.2 µM), the percentage of cells exhibiting early apoptosis increased: 5.72% for Dox, 9.8% for the NPh-Dox-NGR composition.

### 3.7. Effect of Doxorubicin Forms on DNA

The study of DNA–drug interaction by electrochemical methods is based on measurements of the intensity of electrooxidation of guanine, adenine, and thymine by the DPV technique in the presence of drug compositions [35,39].

The interaction of Dox, NPh-Dox, or NPh-Dox-NGR with dsDNA is accompanied by a reduction in DPV oxidation signals of guanine, adenine, and thymine heterocyclic bases, confirming the interaction of the drugs with DNA (Table 2).

**Figure 6:**
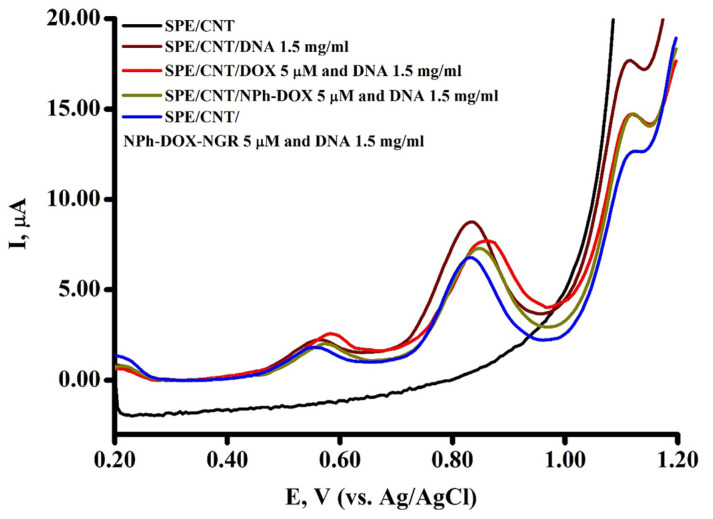
Comparison of the DPV effects of various dosage forms of doxorubicin on DNA on electrodes modified with CNT, where: (-) 1.5 mg/mL DNA, (-) 5 μM Dox and 1.5 mg/mL DNA, (-) 5 µM (NPh-Dox) and 1.5 mg/mL DNA, (-) 5 µM (NPh-Dox-NGR) and 1.5 mg/mL DNA, and (-) SPE/CNT.

As can be observed from Figure 5, there was a positive shift of the differential pulse voltammetry electrooxidation potentials in the presence of 5 μM doxorubicin (Dox) (+0.018, +0.018, and +0.004 V for guanine, adenine, and thymine, respectively). The 5 μM phospholipid nanocomposition of doxorubicin NPh-Dox demonstrated lesser shifts in electrooxidation potentials (+0.008, +0.001, and +0.002 V for guanine, adenine, and thymine, respectively). Positive shifts for Dox and for NPh-Dox may reflect the intercalative mode of interaction of Dox with DNA. This observation is in line with previously published mechanisms of Dox interaction with DNA [35,40]. The negative (cathodic) shifts of potentials for the NPh-Dox-NGR composition (5 μM) are typical for electrostatic interactions due to the charged functional groups of the targeted peptide (−0.002, 0, and 0 V for guanine, adenine, and thymine, respectively) (Figure 6).

Electrochemical platforms represent sensitive and informative approach for the investigation of DNA–drug interaction in vitro or, more correctly, in electrode systems.

It should be noted that this article presents results of the use of therapeutic concentrations of Dox. A more detailed study using various concentrations can be found at [41]. The electrochemical coefficient of the toxic effect of Dox, NPh-Dox, or NPh-Dox-NGR, based on the effect of the oxidation peak of DNA nitrogenous bases on the current, revealed a non-toxic or moderate toxic effect of Dox and Dox nano-derivatives at a concentration range of 0.5–290 µM.

## 4. Discussion

Studies conducted by our laboratory resulted in the incorporation of the chemotherapeutic agent doxorubicin into phospholipid NPs, which led to an increase in its efficacy in vivo [31,42,43]. Further work was aimed at reducing side effects and targeting the phospholipid system of doxorubicin. A peptide containing an NGR with affinity for aminopeptidase N/CD13, which is overexpressed on the surface of a number of tumor cells, was used as a targeted fragment [44,45]. The composition (in addition to the included drug) determines the stability and surface charge, which is largely responsible for the interaction of the resulting drug composition with cells. The size, along with the composition, determines the pharmacokinetics of the particles in the body [12]. In this study, we investigated the physical parameters of the obtained composition of doxorubicin with the targeted fragment (NPh-Dox-NGR). The composition was an ultrathin emulsion with a particle size of not more than 50 nm. The composition without the targeted fragment (NPh-Dox) had a particle size almost two times smaller. Thus, the target conjugated in the form of DSPE-PEG(2000)–NGR logically led to an increase in the particle size. At the same time, this size (less than 50 nm) allowed preservation of the unique properties of nanoparticles, including the ability to avoid clearance by phagocytes of the immune system and maintain long-term blood circulation [46].

Because the electrokinetic Zeta potential (ζ) is an important characteristic, its study will help predict the stability of particles in a solution. The data obtained indicate a more stable state of the composition without the targeted component (more than 15 mV); the introduction of the NGR-containing peptide lowered the value of this parameter. In this regard, the further use of this composition in this form is advisable immediately after preparation; for long-term storage, it requires additional processing, for example, lyophilization, as has been done for a number of other drugs [47,48].

The method of introducing drug delivery systems into the body is determined by their composition, bioavailability, and stability in biological fluids. Since isotonic saline solutions of 0.9% sodium chloride or 5% glucose can be used for intravenous administration, it was important to establish whether an increase in particle size or aggregation will be observed after incubation with the investigated doxorubicin compositions (NPh-Dox and NPh-Dox-NGR). The data obtained indicate a stable state of the composition with and without the targeted fragment in both investigated solutions. Similar data were obtained by other authors studying the properties of doxorubicin NPs modified with folic acid and peptide (RGD) [49].

Of great interest is the sudden release or leakage of the drug into the bloodstream due to the instability of the nanocarrier, which can reduce the therapeutic effect; this is a huge problem for the drug delivery system in vivo. Drug delivery systems that are sensitive to reconstitution due to excellent extracellular stability and rapid intracellular release hold greater potential [50]. The natural pH gradient is known to be 5.0–6.5 in endosomes or lysosomes of tumor cells, and 6.5–7.2 in the tumor microenvironment [36]. Thus, experiments on the release of drugs in vitro simulated the conditions of the release of the drug into the bloodstream and intracellular compartments of tumor cells. The release of doxorubicin from the nanoparticles of the compositions with and without the peptide was shown to occur in a similar way. There was an increase in the level of drug release for up to 5 h of incubation at all pH values, then the level of doxorubicin remained stable for up to 72 h. These results could be due to the protonation of the NH_2_ group of glycine and NH- group of arginine, leading to a break in the bond, which leaves the pores open for Dox to diffuse outward [51,52,53,54]. This property of the doxorubicin phospholipid system could potentially prevent premature release under normal conditions (pH 7.4) and increase its release under acidic conditions (endosomal/lysosomal compartments).

In our previous studies, we showed that the inclusion of the targeted NGR peptide led to an increase in the accumulation of doxorubicin inside the tumor cell (CD13-positive) (internalization) [32]. However, we did not conduct additional studies to evaluate and understand the possible mechanism of action of the developed composition. The studies performed in this work on two cell lines, HT-1080 and MCF-7 (expressing and not expressing aminopeptidase N, respectively, shown using a Western blot [48]), indicated a direct dependence of the inhibitory effect on the dose of the drug used and the presence of the targeted peptide in the system. In the variant with CD13-positive HT-1080 cells, the degree of cytotoxic action of the composition variants containing the targeted peptide significantly exceeded CD13-negative MCF-7 cells. At the maximum concentration (25 µM), 100% cell death was observed after 24 h for both compositions, with and without the peptide. The data obtained confirms the differences we previously observed in the total accumulation and internalization on the same cells, depending on the presence of a specific peptide containing the NGR in the composition. There was an almost twofold increase in the total accumulation of Dox in HT-1080 cells compared to the phospholipid form of Dox (NPh-Dox). There was also an increase in Dox penetration with the targeted conjugate in the delivery system [32]. We obtained similar results with the chlorin e6 phospholipid composition in an experiment with two cell lines (HepG2 and MCF-7) [55].

Several studies have reported that doxorubicin induces apoptosis by activating caspases and disrupting the mitochondrial membrane potential [56,57]. A study of the cell death pathway depending on incubation both with the phospholipid system itself and with its components separately showed that death was predominantly apoptotic in all variants. Introducing the targeted NGR-containing peptide into the phospholipid composition did not lead to a change in the pathways of tumor cell death; however, an increase in the concentration of doxorubicin in the compositions and the free form to 2 µM led to an increase in the percentage of cell necrosis (not more than 25%). Interesting results were obtained in a study [58] where 24 h treatment of cells with doxorubicin and poly-L-arginine caused a slight increase in doxorubicin-induced apoptosis and significantly increased necrosis in DU145 cells. In our variant, the NGR peptide clearly increased the percentage of apoptotic cells only in the variant with the maximum used concentration of doxorubicin (2 µM) by 20% compared to its phospholipid form without the peptide. This effect may be due to the high degree of internalization and intracellular release of the cytostatic agent due to the targeted NGR peptide used.

Label-free electrochemical DNA sensing of heterocyclic nucleobases is an attractive approach for DNA registration itself and for investigation of drug–DNA complex formation [4,5,6]. The mechanism of interactions between medicinal therapeutic drugs with DNA can be assessed by the shift in the electrooxidation potential of guanine, adenine, and thymine. Shifts of potentials to the positive (anodic) direction reflect the intercalation mechanism due to the difficulty of electron flow to or from electro oxidized fragment; a shift in the negative (cathodic) direction means the formation of hydrogen bonds and/or electrostatic interactions with DNA molecule [4,5,6]. In our study, there was a shift of electro-oxidation potentials towards the positive in the case of Dox and NPh-Dox, which is typical for intercalative mode. Electrostatic interactions due to the charged functional groups of the targeted peptide were registered for the peptide-conjugated form of Dox (NPh-Dox-NGR), possessing charged groups of peptide residues.

Thus, the study demonstrated the main properties of the resulting phospholipid composition of doxorubicin with the targeted NGR-containing peptide, as well as its benefits compared to the free drug and its phospholipid composition without the targeted component. The data obtained confirm the significance of aminopeptidase N/CD13 expression and the possibility of using this protein as a target for targeted delivery. The results of this study may be necessary for further preclinical evaluation of the obtained composition.

## 5. Conclusions

The developed compositions of Dox embedded in phospholipid NPs or NPs with NGR are ultrathin emulsions with a particle size of less than 50 nm. There were no changes in particle size during incubation in isotonic solutions (glucose and NaCl), which indicated the stability of the system. An in vitro study of the release of Dox from NPs showed a dependence on pH. The release percentage increases with a more acidic pH, which may increase the efficacy of Dox release in intracellular compartments (endosomes/lysosomes). The assessment of the cytotoxic effect of the compositions showed a direct dependence on the dose of the drug used, the presence of the targeted component in the composition, and the time of incubation with the substances. The differences included increased cell proliferation during incubation with CD13-positive (HT-1080) cells and decreased proliferation with CD13-negative cells. This effect is probably associated with different levels of penetration into the cell, which was confirmed by our earlier study. Cell death pathway study has shown that death occurred mainly by apoptosis. The inclusion of the targeted NGR peptide (NPh-Dox-NGR) increased the percentage of cells undergoing apoptosis at the maximum drug concentration.

The intercalative mechanism of Dox and NPh-Dox was confirmed by electrochemical technique, based on DPV signals of nucleobases in the presence of drugs.

Electrostatic interactions with dsDNA due to the charged functional groups of the targeted peptide (NH2-)Gly-Asn-Gly-Arg-Gly-Cys(-COOH) were registered for peptide conjugated form of Dox (NPh-Dox-NGR), possessing additional charged groups of peptide residues.

Thus, the results obtained are interesting and require further experimental studies for a more detailed clarification of the mechanism of action of the developed composition with targeted peptide NGR as a directed component of a drug.

## Figures and Tables

**Table 1:** Change in particle size and distribution of NPh-Dox and NPh-Dox-NGR compositions after incubation in isotonic solutions (5% glucose and 0.9% NaCl) at 37 °C.

Samples		Incubation Time, h							
		0	0.25	0.5	1	3	24	48	72
0.9% NaCl									
NPh-Dox	nm	33.73 ± 1	27.92 ± 2.2	28.22 ± 1.9	28.67 ± 1.4	28.87 ± 1	30.18 ± 2.2	31.9 ± 1.1	29.94 ± 1.7
	%	99.9 ± 0.1	99.67 ± 0.2	99.73 ± 0.1	99.7 ± 0.2	99.7 ± 0	96.9 ± 0.8	97.47 ± 0.4	96.57 ± 0.7
NPh-Dox-NGR	nm	36.95 ± 5.2	35.39 ± 3.2	36.41 ± 0.5	34.21 ± 0.3	35.17 ± 0.7	36.47 ± 1.6	37.12 ± 0.8	36.92 ± 2
	%	99.7 ± 0.5	100 ± 0	95.23 ± 8.3	99.87 ± 0.2	100 ± 0	100 ± 0	99.77 ± 0.2	92.47 ± 12.7
5% Glucose									
NPh-Dox	nm	18.07 ± 0.9	15.99 ± 0.8	15.8 ± 0.9	15.34 ± 2.1	16.26 ± 2	16.24 ± 0.3	18.81 ± 1.2	18.29 ± 1.6
	%	99.97 ± 0.1	99.9 ± 0	100 ± 0	100 ± 0	99.97 ± 0.1	100 ± 0	100 ± 0	100 ± 0
NPh-Dox-NGR	nm	33.28 ± 3.8	34.76 ± 2.2	33.64 ± 4.7	34.82 ± 0.7	34.88 ± 3.9	33.9 ± 4.6	34.05 ± 3.1	34.74 ± 3.2
	%	91 ± 15.6	100 ± 0	100 ± 0	99.9 ± 0.2	100 ± 0	100 ± 0	100 ± 0	95.77 ± 7.3

**Table 2:** The ratio of DPV oxidation current intensities I(DNA+drug)/I(DNA) for 5 μM drug concentration, calculated for guanine (G), adenine (A), and thymine (T) heterocyclic bases.

Drug-DNA Complex	G	A	T
Dox	0.88	0.81	0.77
NPh-Dox	0.77	0.84	0.78
NPh-Dox-NGR	0.85	0.84	0.62

## Data Availability

The data are available on reasonable request from the corresponding author.
